# Nonresonant Polarized Raman Spectra Calculations of Nitrogen-Doped Single-Walled Carbon Nanotubes: Diameter, Chirality, and Doping Concentration Effects

**DOI:** 10.1155/2020/1409581

**Published:** 2020-04-27

**Authors:** F. Allali, H. Chadli, M. Bentaleb, P. Hermet, A. Rahmani

**Affiliations:** ^1^Laboratoire d'Etude des Matériaux Avancés et Applications (LEM2A) Université Moulay Ismaïl, FSM-ESTM, BP 11201, Zitoune, Meknes, Morocco; ^2^Institut Charles Gerhardt Montpellier, UMR-5253, CNRS, Université de Montpellier, ENSCM, Place E. Bataillon, 34095 Montpellier, France

## Abstract

Raman spectra of nitrogen-doped single-walled carbon nanotubes are calculated using the spectral moment's method combined with the bond polarizability model. The influence of the nanotube diameter and chirality is investigated. We also address the important question of the effect of the *N*-doping concentration, and we propose an equation to estimate the doping concentration from the knowledge of the tube diameter and the frequency of the radial breathing mode.

## 1. Introduction

Since their first observation in 1991 by Iijima [1], carbon nanotubes (CNTs) have drawn enormous attention of both experimentalists and theoreticians. Single-walled carbon nanotubes (SWCNTs) have become a standard material in nanotechnology due to their exceptional physical properties. In order to unlock the potential of applications of these nanomaterials in the field of energy, many doping processes are considered (intercalation and substitution for example) [2–7]. The properties of SWCNTs are mainly dependent on the tubular diameter and chirality which make their synthesis for the specific purposes difficult. They can also be either metallic or semiconducting [8]. A modification of SWCNTs properties, by controlling placing heteroatoms, leads to enormous technological implications which justify the number of experimental and theoretical investigations focused on this topic. Thus, the electronic properties of SWCNTs can be modified by manipulating the Fermi level, which can be done by chemical doping.

It was experimentally and theoretically found that the introduction of heteroatoms such as boron or nitrogen into SWCNTs can efficiently tailor their structural and electronic properties [7, 9–15], which might lead to a wider range of applications [16–18]. Recently, the nanotubes are produced by in situ nitrogen substitutional doping, and they are used singularly or in multiples, for example, in molecular electronics, power transmission cables, batteries, solar cells, probes and manipulators, and composites [19, 20].

Heteroatom-doped carbon nanomaterials have attracted significant attention as anode materials for sodium-ion batteries [20, 21], SWCNTs and nitrogen-doped SWCNTs as emergent nanomaterials for many applications can be synthesized by different techniques, such as laser ablation, arc-discharge, chemical vapor deposition (CVD), and spray pyrolysis. CVD method can be categorized into thermal-assisted [22–24] and plasma-assisted [25] processes. The growth of carbon materials by plasma CVD tends to have an individually and vertically free-standing due to the strong electric fields in plasma sheaths [25, 26].

Recently, great attention has been devoted to the study of nitrogen-doped carbon nanotubes also because of their excellent electrical conductivity and electrocatalytic activity for oxygen reduction reaction [22, 23, 27–35]. Several studies have been experimentally carried out on the synthesis of CNTs and nitrogen-doped carbon nanotubes [36] by using different precursors such as caffeine [37], melamine, and nickel acetate [38]. Thus, benchmark theoretical Raman spectra of *N*-doped SWCNTs (*N*-SWCNTs) as a function of their structural parameters (diameter, chirality, and atom *N*% concentration) are important to understand experimental data [3, 39].

Raman spectroscopy is the most used method by experimentalists to identify the type of nanotubes and to study their vibrational and electronic properties [36, 40, 41]. The Raman spectrum of SWCNT is dominated by *A*1 g radial breathing modes (RBM) below 500 cm^−1^, and by tangential modes (TM) in the high-frequency region (1400–1600 cm^−1^) [39, 41]. The latter consist of two *A*, two *E*1, and two *E*2 phonon modes for chiral nanotubes, and one *A*1 g, one *E*1 g, and one *E*2 g mode for achiral (armchair and zigzag) nanotubes. It was found that the RBM frequency closely depends on the diameter of the tubes, regardless of their chirality [42]. The TM shape, however, depends strongly on whether the tube is metallic or semiconducting [43].

Calculated Raman spectra of *N*-doped SWCNTs can be useful to estimate the effect of diameter and doping level of nanotubes by analysis of the frequency shift and scattering intensity of Raman spectra modes. Vibrational and electronic properties of those nanomaterials have been calculated by different approaches: ab initio calculations [44, 45], density functional theory [9, 46], and tight-binding approach [47, 48]. Recent research revealed that N-SWCNT shows improvement for energy storage applications [49, 50]. Other results indicated that the nitrogen-doped bamboo-like carbon nanotubes encapsulated with nickel nanoparticles can show emerging functionalities for environmental remediation processes [51].

In this paper, we report calculated polarized Raman spectra of N-SWCNTs as a function of their diameter and chirality. We also address the important question of the effect of the N-doping concentration, and we propose an equation derived for our calculations to estimate the doping concentration from Raman measurements. For these purposes, we optimized a force-field model derived from the Saito potential to build the dynamical matrix, and the Raman susceptibility of modes was calculated using the bond polarizability mode. Because we interested in systems which are large and have low symmetry, we used the spectral moment's method [52] to avoid diagonalization of the dynamical matrix.

## 2. Models and Method

The SWCNT structure can be uniquely specified by the pair of integers *n* and *m*. The chiral angle, *θ*, is defined as the angle between the chiral vector, $\overset{⟶}{C_{h}}$, and the zigzag direction (*θ*=0). For an ideal tube, the radius *R*, translation vector *T*, and chiral angle are given by the following relations: $R=a\sqrt{3\left(n^{2}+m^{2}+nm\right)}/2\pi$, $T=2\sqrt{3\pi }\left(R/d\right)$, and $\theta =\arctan\left(\sqrt{3m/m+2n}\right)$, where *a* is the lattice constant of 2*D* graphene sheet and *d* is the greatest common divisor of 2*n*+*m* and 2*m*+*n*. The number of hexagons, *N*, contained within the 1*D* unit cell of a nanotube is determined by *N*=2(*m*^2^+*n*^2^+*nm*)/*d*.

**Figure 1:**
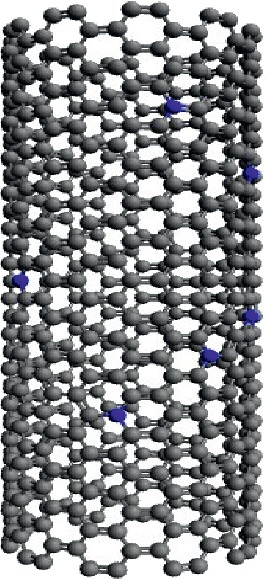
Geometric structure of direct substitution of six nitrogen atoms into the carbon atoms in *C*_94_*N*_6_.

Nitrogen-doped SWCNTs (N-SWCNTs) have been built substituting randomly carbon atoms inside SWCNT by nitrogen atoms. We fixed in the random sampling a condition to have a *N*-*N* distance greater than 0.48 nm. A schematic representation of the generated structure of *N*-SWCNTs is shown in Figure 1. The doping concentration, *τ*, is defined by the nitrogen atomic percent (%) which gives the percentage of nitrogen atom relative to the total number of atoms in the tube.

The force constant model of Saito [42] is a force-field model well known inside the nanotube community. This model consists in the direct parametrization of the diagonal real-space force constants including up to fourth nearest-neighbor interactions. This leads to a set of 12 adjustable parameters which are presently known only for carbon atoms and gives excellent predictions on the phonon frequencies in SWCNTs. In the case of *N*-SWCNTs, we chose this force field model to compute the dynamical matrix (leading to the frequency of the Raman modes). The *C*-*N* interactions have been parametrized by fitting the phonon modes in the (5, 5) *N*-SWCNT with 5% nitrogen dopants calculated by density functional theory (DFT) [53]. DFT calculations on the (5, 5) *N*-SWCNT with 5% nitrogen dopants have been performed using the SIESTA package [54] and the local density approximation to the exchange correlation functional as proposed by Ceperley and Alder [55]. The valence electrons were described by a double-zeta singly polarized basis set. The localization of the basis is controlled by an energy shift of 50 meV. Real space integration is performed on a regular grid corresponding to a plane-wave cutoff around 350 Ry. 31 *k*-points along the nanotube axis have been used. Atomic positions were relaxed using a conjugate gradient until the maximum residual atomic force was smaller than 0.02 eV/Å. The vacuum size was fixed to 11 Å. Zone-center phonons were calculated within the harmonic approximation by finite difference of the Hellmann–Feynman forces with an atomic displacement of 0.03 Å. Positive and negative displacements are used to minimize anharmonic effects.

The *C*-*C* interactions have been fixed to the initial values given by Saito at the beginning of the fit [42]. The 24 optimized force constants used in the calculation of the dynamical matrix in *N*-SWCNTs are listed in Table 1. Note that, in the chosen geometry of *N*-SWCNTs, the interatomic *N*-*N* distance exceeds 0.48 nm, and consequently, the *N*-*N* interactions are neglected in our calculations.

The nonresonant Raman scattering spectra have been calculated within the framework of the bond polarizability model [56]. In this case, the polarization is only modulated by the nearest-neighbor bonds, and the components of the induced polarizability tensor $\tilde{\pi }$ are given by the empirical equation [57, 58]:

(1) $$\begin{array}{c} \pi_{\alpha \beta }\left(r\right)=\frac{1}{3}\left(\alpha_{l}+2\alpha_{p}\right)\delta_{\alpha \beta }+\left(\alpha_{l}-\alpha_{p}\right)\left({\hat{r}}_{\alpha }{\hat{r}}_{\beta }-\frac{1}{3}\delta_{\alpha \beta }\right), \end{array}$$

where *α* and *β* relate to the Cartesian components (*x*, *y*, *z*) and $\overset{⟶}{\hat{r}}$ is the unit vector along the vector $\overset{⟶}{r}$ connecting atom *n* and atom *m* which are covalently bonded. The parameters *α*_*l*_ and *α*_*p*_ correspond to the longitudinal and perpendicular bond polarizability, respectively. Within this approach, one can assume that the bond polarizability parameters are functions of the bond lengths *r* only.

The coefficients *π*_*αβ*,*γ*_^*n*^ connect the polarization fluctuations to the atomic motions, and they are obtained by expanding the polarizability tensor *π*^*n*^ in terms of the *n*^th^ atomic displacements. The derivatives *π*_*αβ*,*γ*_^*n*^ are given by

(2) $$\begin{array}{c} \pi_{\alpha \beta ,\gamma }^{n}=\sum_{m}\frac{1}{3}\left(2\alpha_{p}^{\prime }+\alpha_{l}^{\prime }\right)\delta_{\alpha \beta }{\hat{r}}_{\gamma }+\left(\alpha_{l}^{\prime }-\alpha_{p}^{\prime }\right)\left({\hat{r}}_{\alpha }{\hat{r}}_{\beta }-\frac{1}{3}\delta_{\alpha \beta }\right){\hat{r}}_{\gamma }+\frac{\left(\alpha_{l}-\alpha_{p}\right)}{r}\left(\delta_{\alpha \gamma }{\hat{r}}_{\beta }+\delta_{\beta \gamma }{\hat{r}}_{\alpha }-2{\hat{r}}_{\alpha }{\hat{r}}_{\beta }{\hat{r}}_{\gamma }\right), \end{array}$$

where *α*′=(∂*α*/∂*r*)|_*r*=*r*_0__ and *r*_0_ is the equilibrium bond distance. Thus, the bond polarizability (BP) model of *N*-CNT nanotubes is completely defined by three parameters: $\bar{\alpha }=2\alpha_{p}^{\prime }+\alpha_{l}^{\prime }$ , $\bar{\beta }=\alpha_{l}^{\prime }-\alpha_{p}^{\prime }$, and $\bar{\gamma }=\left(\alpha_{l}-\alpha_{p}\right)/r$ for each type of bond (*C*-*N* and *C*-*C*).

These BP parameters are usually fitted to reproduce the experimental Raman spectrum. However, they have been determined in our work from density functional perturbation theory on the *h* − *CN* sheet following the procedure described in Ref. [59]. The obtained values are listed in Table 2. Here, we suppose the transferability of the BP parameters obtained for the *h* − *CN* to predict the Raman spectra of nitrogen-doped single-wall carbon nanotubes.

When the system contains a large number of atoms, as for long tubes of finite length, $\tilde{D}$ is very large, and its diagonalization fails or requires long computational time. By contrast, the spectral moment's method (SMM) allows to compute directly the Raman spectra of very large harmonic systems without any diagonalization of $\tilde{D}$. In this case, the frequencies of the Raman bands are directly known from the position of bands in the calculated spectra. Of course, both approaches lead exactly to the same band positions and relative intensities for small samples.

The SMM consists of developing the resolvent *R*(*z*) associated with the response function into a continued fraction [52, 60]:

(3) Ju=−1πlimε⟶0+ImRz,

where *z* = *u* + *iε* and

(4) $$\begin{array}{c} R\left(z\right)=\frac{b_{0}}{z-a_{1}-\left(b_{1}/z-a_{2}-\;\vdots \right)}. \end{array}$$

The coefficients *a*_*n*_ and *b*_*n*_ are given by

(5) $$\begin{array}{c} a_{n+1}=\frac{{\bar{\nu }}_{n}}{\nu_{n}},b_{n}=\frac{\nu_{n}}{\nu_{n-1}}. \end{array}$$

The spectral generalized moments *ν*_*n*_ and ${\bar{\nu }}_{n}$ of *J*(*u*) are directly obtained from $\tilde{D}$. Each element of $\tilde{D}$ is given by

(6) $$\begin{array}{c} D_{\alpha \beta }\left(\kappa ,\kappa^{\prime }\right)=\frac{1}{\sqrt{m_{\kappa }m_{\kappa^{\prime }}}}\phi_{\alpha \beta }\left(\kappa ,\kappa^{\prime }\right), \end{array}$$

where *ϕ*_*αβ*_(*κ*, *κ*′) is the interatomic force constant matrix between atoms *κ* and *κ*′.

## 3. Results and Discussion

In this section, we report the polarized Raman spectra calculated for isolated *N*-SWCNTs of different diameters using SMM. The polarized Raman spectroscopy experiments on aligned SWNTs show a simple intensity dependence of the signal: both the RBM and TM intensities peak at a maximum when the light is polarized along the tube axis and are significantly reduced when the light (either incident or scattered) is polarized perpendicular to the tube axis. Previously, we have calculated the polarized nonresonant Raman spectra of SWCNTs of different lengths [39]. The calculation of the polarized XY spectrum is useful for the interpretation of the experimental results since it makes it possible to determine the symmetries of the modes.

In our working method, the frequency of modes is directly obtained from the peak position in the calculated spectrum. The line shape is assumed in our calculation to be Lorentzian, and the line width is fixed at 1.7 cm^−1^. In all our calculations, the *Z*-direction is considered as the nanotube axis direction. We mainly discuss the *ZZ*-polarized Raman spectra for which the polarization direction of the incident and scattered light are along the *Z*-direction.

### 3.1. Polarized Raman Spectra of *N*-SWCNT Inifinite Crystals

**Figure 2:**
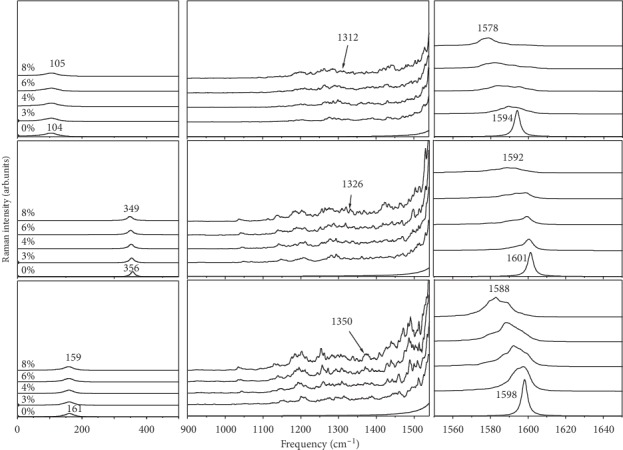
Raman spectra in (10, 10) *N*-SWCNT as a function of the nitrogen concentration (in %) calculated for a *ZX* (top), *YX* (middle), and *ZZ* (bottom) polarization. All the spectra are displayed with the same intensity scale.

**Figure 3:**
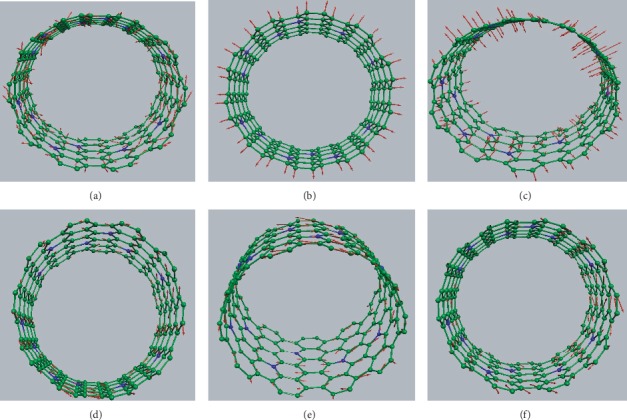
Calculated atomic motions (eigen displacement vectors) in (10, 10) *N*-SWCNT with 8% nitrogen dopant. Arrows are proportional to the amplitude of the atomic motions, (a) 105 cm^−1^, (b) 159 cm^−1^, (c) 349 cm^−1^, (d) 1578 cm^−1^, (e) 1588 cm^−1^, and (f) 1592 cm^−1^.

We have calculated the polarized Raman spectra of an armchair *N*-doped (10, 10) as a function of nitrogen doping (1–8%). Results are reported in Figure 2 and compared with the Raman spectra of an undoped (10, 10) SWCNT for the *ZX*, *YX*, and *ZZ*-polarization. We observe that these spectra can be divided into three frequency regions: (i) a low-frequency region below 500 cm^−1^ where the breathing mode dominates, (ii) an intermediate-frequency region between 500 and 1500 cm^−1^, and (iii) a high-frequency region above 1500 cm^−1^ where the tangential modes are located. The intensities of the different polarized spectra have been normalized and can be compared. The atomic motions (eigen displacement vectors) of the main Raman lines in the (10, 10) *N*-SWCNT with 6% *N* are displayed in Figure 3. For the purpose of this figure, the direct diagonalization of the dynamical matrix has been used instead of the spectral moment's method in order to have access to the eigen displacement vectors.

In the low-frequency range, each Raman spectrum is featured by a single peak assigned to the *E*_1*g*_, *E*_2*g*_, and *A*_1*g*_ radial modes of undoped (10, 10) tube in the *ZX*, *YX*, and *ZZ* polarization, respectively. A small downshift (3–5 cm^−1^) of the two last modes is observed when the nitrogen atoms are substituted in the tube, whereas the first one seems unaffected with the doping. For instance, *A*_1*g*_ of the undoped (10, 10) SWCNT shifts from 161 cm^−1^ in pure (10, 10) to 159 cm^−1^ in (10, 10) with 8% nitrogen concentration. The eigen displacement vectors displayed in Figure 3(b) show that this mode has the characteristic of the radial breathing mode (RBM): all carbon and nitrogen atoms move in phase in the radial direction, and the mode is fully symmetric. Similarly, the *E*_2*g*_ mode of undoped (10, 10) tube downshifts to 349 cm^−1^ in *N*-doped tube (Figure 2) and corresponds to a mixing of tangential and radial displacement of *C* and *N* atoms (Figure 3(c)). The line centered at 105 cm^−1^ is a longitudinal mode as seen in Figure 3(a).

In the intermediate region, which corresponds to the location of the *D*-band in SWCNTs, no mode is observed in undoped tube for the three types of polarization. In contrast, this region shows, as illustrated by Figure 2, a very complex multiband structure with the doping as several lines can be evidenced whatever the polarization. In the *ZZ* and *YX* polarizations, the spectra of *N*-doped (10, 10) tube are dominated by three broad bands near 1240, 1320, and 1340 cm^−1^.

In the high-frequency range, the Raman spectra of the undoped (10, 10) tube are dominated by a strong Raman line whatever the polarization. This line is called the *G*-band in the literature. It is located at 1594 (*E*_1*g*_), 1601 cm^−1^ (*E*_2*g*_), and 1598 (*A*_1*g*_), in the *ZX*, *YX*, and *ZZ* polarization, respectively. With the nitrogen doping, our calculations state that, in comparison with the undoped tube, the profile of these lines are broader, and their positions show a significant downshift (∼10 cm^−1^), and they are, respectively, around 1578, 1592, and 1588 cm^−1^ in the (10, 10) *N*-SWCNT with 8% *N*. Concerning the atomic motions, atoms vibrate along the tangential (resp., longitudinal) direction for the mode centered at 1578 (resp., 1592) cm^−1^ (Figures 3(d) and 3(f)). The mode centered at 1588 cm^−1^ is a totally symmetric tangential mode (Figure 3(e)) similar to the *A*_1*g*_ mode in the undoped tube. The study of the chirality dependence in the high-frequency region should be promising to investigate properties where lattice effects are more pronounced.

### 3.2. Chirality Effect

**Figure 4:**
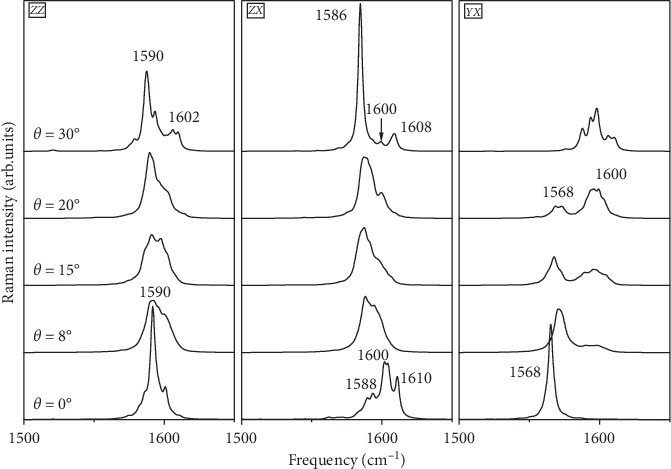
Chirality dependence of *N*-SWCNT with 6% nitrogen dopant on polarized Raman spectra.

The chirality dependence of the Raman spectra have been calculated on an infinite *N*-SWCNT with 6% nitrogen dopant and considering five values of the chiral angle *θ* = 0, 8 15, 20, and 30°. These angles are associated to (17, 0), (17, 3), (14, 5), (14, 7), and (10, 10) nanotubes, respectively. Calculated spectra are shown in Figure 4 within the tangential modes region and for a *ZZ*, *ZX*, and *YX* polarization. Similarly to our previous calculations on undoped SWCNTs [39], we found that the tangential modes are especially the most sensitive to the chirality, while the low-frequency modes are mainly sensitive to the tube diameter.

We observe that the *ZZ*- and *YX*-polarized Raman spectra in the zigzag *N*-SWCNT (*θ*=0°) are dominated by a single band with a complex multistructure centered in the 1590 cm^−1^ and 1568 cm^−1^, respectively. The increase of the chirality from 0 to 30° leads to the appearance of a second band, with also a complex multistructure, at a frequency higher than that of the first one. For instance, this band is centered around 1600 cm^−1^ in the both polarizations. In the *ZX* polarization, the tangential armchair and zigzag tubes are characterized by three lines located around 1587, 1600, and 1609 cm^−1^. These lines change in intensity and overlap, leading to a large band centered around 1590 cm^−1^ in achiral tubes.

### 3.3. Diameter and Doping Concentration Dependence

In real doped carbon nanotube samples, the exact degrees of nitrogen concentration ranges are not really known, but it can range up to 10%. Thus, we tried to derive a model to estimate this concentration from Raman spectra. However, using the SMM, we are not able to calculate the dependence of the absolute intensity of each mode with the excitation energies as we combine here the spectral moment's method and the nonresonant bond polarizability model.

**Figure 5:**
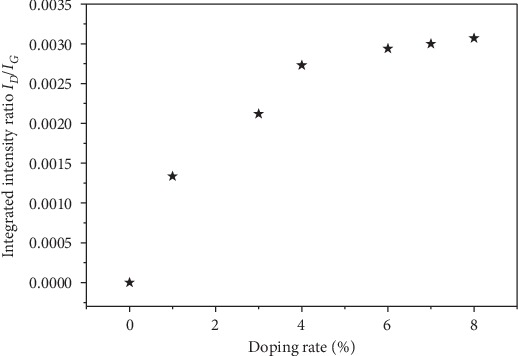
Integrated intensity ratio, *I*_*D*_/*I*_*G*_, as a function of nitrogen concentration.

We considered armchair and zigzag *N*-SWCNT whose diameters vary up to 6 nm. For each diameter, the low-frequency Raman lines have been calculated as a function of the nitrogen doping concentration. We focus on the *ZZ*-polarized Raman spectrum (Figure 2). The spectra were calculated for nine values of the nitrogen concentration, and the integrated intensity ratio between the *G*-band located around 1590 cm^−1^ (*I*_*G*_) and the *D*-band located around 1340 cm^−1^ (*I*_*D*_) is calculated. The obtained *I*_*D*_/*I*_*G*_ ratio as function of the nitrogen concentration in *N*-(10, 10) nanotube is displayed in Figure 5. As expected, it is obvious that the increase of nitrogen atoms in carbon nanotube causes an increase of the *I*_*D*_/*I*_*G*_ ratio. This suggests a possible loss of ordering accruing in the nanotube surface as more nitrogen is substituted into the carbon nanotube. This behavior of the *I*_*D*_/*I*_*G*_ ratio with the increase of nitrogen concentration is consistent with the observations of Stephen and Ariharan [36, 61].

We have also investigated the dependence of some specific Raman active modes of *N*-SWCNTs as a function of the diameter of the nanotube. The dependencies of the frequencies of these modes as a function of the diameter are similar to those found in undoped SWCNTs [39]. We already reported that the low-frequency modes (lower than 500 cm^−1^) are the most influenced mode by the nanotube diameter especially the RBM mode. This mode is the most appropriate for extracting structural and dynamical information of SWCNTs.

We have previously found [3, 39], using the bond polarizability model combined with the spectral moments method, a relation between diameter (*D*) and RBM frequency (*ω*): *ω*=*A*/*D* for isolated undoped nanotubes [62] (doping concentration *τ*=0%), where the parameter *A*=217; 146 and 484 nm. cm^−1^ for RBM, *E*_1*g*_ and *E*_2*g*_, respectively.

**Figure 6:**
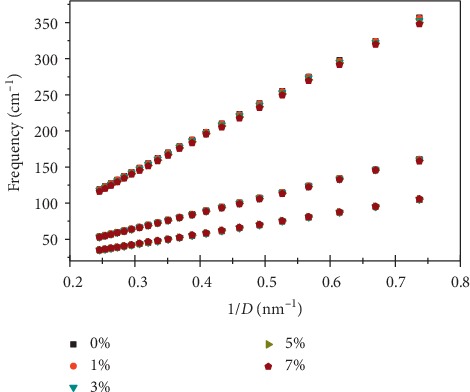
Calculated frequency dependence of the radial *A*_1_, *E*_1_, and *E*_2_ modes as function of nitrogen concentration (0 ≤ *τ* ≤ 8%) and tube diameter (1.35 ≤ *D* ≤ 4.00 nm) in armchair *N*-SWCNTs.

Figure 6 shows the evolution of RBM, *E*_1_ and *E*_2_ modes' frequencies as a function of diameter (1.35–4 nm) for a doping concentration *τ* ranging from 0 to 8%. Globally, and for a given polarization, the dependence on the mode frequencies is similar with diameter. For the three modes, the evolution of the Raman active modes in undoped SWCNT is qualitatively the same as those in N-SWCNT (*τ*>0%): their frequencies decreases with the tube diameters' increase. In contrast with the significant dependence of the Raman modes frequencies with tube diameter, the lines are slightly dependent on the nitrogen concentration, and all Raman modes downshift when the nitrogen concentration increases. We found that, for each nitrogen concentration, the frequency of these modes follows a law in *A*/*D*. The calculated *A*-parameter values are listed in Table 3 for four values of nitrogen concentration *τ*=2,4,6, and 8%.

**Figure 7:**
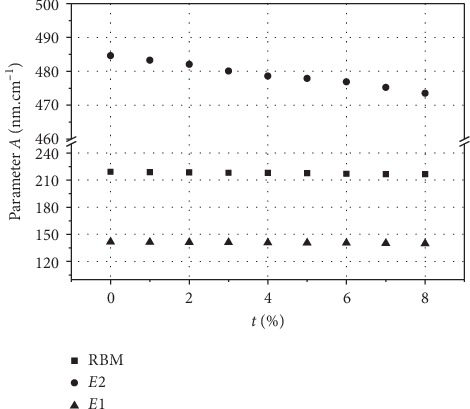
Dependence of the parameter *A* as a function of the doping concentration *τ*.

Then, the evolution of the calculated *A*-parameter values as a function of nitrogen concentration is displayed in Figure 7 for the three Raman lines below 350 cm^−1^: RBM, *E*_1*g*_ and *E*_2*g*_. From these results, we get a phenomenological relation between the diameter *D*, the doping concentration *τ* and the frequency of the RBM-like mode according to: *ω*=(*A* − *C*.*τ*)/*D*, where the *A* and *C* parameters are listed in Table 4. We highlight that this equation could be useful to estimate the diameter and the nitrogen concentration in N-SWCNTs from the experimental Raman spectra.

## 4. Conclusion

In this paper, we have first computed the polarized Raman spectra in *N*-SWCNTs as a function of their diameter and chirality. The most significant changes of the Raman spectrum occur in the high-frequency range, which indicates the effect of the doping. Then, the study according to the nitrogen concentration allowed us to make the ratio of the integrated intensities for *D* and *G* bands as a function of doping rate. We derived a phenomenological equation describing the diameter dependence on the radial low-frequency Raman modes below 500 cm^−1^. The latter region is also especially sensitive to the tube diameter and the nitrogen doping concentration. We found that the behavior of the RBM mode with the tube diameter was clearly modified in *N*-SWCNT. This involves that the linear relation between the RBM frequency and the inverse of the tube diameter found in undoped SWCNT has to be modified in the case of *N*-doped SWCNTs sample. Finally, we think that the calculated Raman spectra reported in the present work can be useful to understand Raman data and estimate the nitrogen concentration.

## Figures and Tables

**Table 1:** Optimized force constants according to the nomenclature given by Saito (×10^4^ dyn/cm).

Radial	Tangential	
*ϕ*_*r*_^1(*C* − *C*)^=360	*ϕ*_ti_^1(*C* − *C*)^=240	*ϕ*_to_^1(*C* − *C*)^=92
*ϕ*_*r*_^1(*C* − *N*)^=315	*ϕ*_ti_^1(*C* − *N*)^=172	*ϕ*_to_^1(*C* − *N*)^=74
*ϕ*_*r*_^2(*C* − *C*)^=90	*ϕ*_ti_^2(*C* − *C*)^=−56	*ϕ*_to_^2(*C* − *C*)^=−6.6
*ϕ*_*r*_^2(*C* − *N*)^=102	*ϕ*_ti_^2(*C* − *N*)^=−41	*ϕ*_to_^2(*C* − *N*)^=−4.8
*ϕ*_*r*_^3(*C* − *C*)^=35.1	*ϕ*_ti_^3(*C* − *C*)^=−24.4	*ϕ*_to_^3(*C* − *C*)^=2.7
*ϕ*_*r*_^3(*C* − *N*)^=−27.5	*ϕ*_ti_^3(*C* − *N*)^=15.9	*ϕ*_to_^3(*C* − *N*)^=−1.5
*ϕ*_*r*_^4(*C* − *C*)^=−22.4	*ϕ*_ti_^4(*C* − *C*)^=19.3	*ϕ*_to_^4(*C* − *C*)^=−5.6
*ϕ*_*r*_^4(*C* − *N*)^=−16.9	*ϕ*_ti_^4(*C* − *N*)^=18.9	*ϕ*_to_^4(*C* − *N*)^=−9.7

**Table 2:** Optimized bond polarizability parameters of adjusted model for each type of bond.

Bond	$\bar{\alpha }\left({Å}^{2}\right)$	$\bar{\beta }\left({Å}^{2}\right)$	$\bar{\gamma }\left({Å}^{3}\right)$
*C*-*C*	4.7	4	0.04
*C*-*N*	−6.53	0.9	0.55

**Table 3:** Values of the *A*-parameter (in nm. cm^−1^) as function of the nitrogen concentration *τ*.

*τ*(%)	2			4			6			4		
Mode	*A*_1_	*E*_1_	*E*_2_	*A*_1_	*E*_1_	*E*_2_	*A*_1_	*E*_1_	*E*_2_	*A*_1_	*E*_1_	*E*_2_
*A*	218.5	141.3	482.1	217.9	140.8	478.6	217	140.4	476.9	216.4	140.1	473.5

**Table 4:** Values of the *A* and *C* parameters according to *ω*=(*A* − *C*.*τ*)/*D* low.

Mode	*A*_1_	*E*_1_	*E*_2_
*A* (nm. cm^−1^)	219.15	141.7	484.54
*C* (nm. cm^−1^)	0.34	0.26	1.35

## Data Availability

All data used to support the findings of this study are included within the article. The data analyzed during the current study are available from the corresponding author upon reasonable request.

## References

[1] Iijima S. (1991). Helical microtubules of graphitic carbon. *Nature*.

[2] Noelia A., Ignacio M.-G., Rafael F., Francisca Gomez-Rico M. (2018). Production of bamboo-type carbon nanotubes doped with nitrogen from polyamide pyrolysis gas. *Journal of Analytical and Applied Pyrolysis*.

[3] El Biyaali A., Bentaleb M., Rahmani A. H. (2015). Tube-length dependence on theoretical Raman spectra of single-walled BC3 nanotubes and bundle size effect. *The Journal of Physical Chemistry C*.

[4] Weng-Sieh Z., Cherrey K., Chopra N. G. (1995). Synthesis of BxCyNz nanotubules. *Physical Review B*.

[5] Terrones M., Hsu W. K., Terrones H. (1996). Metal particle catalysed production of nanoscale BN structures. *Chemical Physics Letters*.

[6] Suenaga K., Colliex C., Demoncy N., Loiseau A., Pascard H., Willaime F. (1997). Synthesis of Nanoparticles and nanotubes with well-separated layers of boron nitride and carbon. *Science*.

[7] Stephan O., Ajayan P. (1994). Doping graphitic and carbon nanotube structures with boron and nitrogen. *Science*.

[8] Saito R., Fujita M., Dresselhaus G., Dresselhaus M. S. (1992). Electronic structure of chiral graphene tubules. *Applied Physics Letters*.

[9] Mohsen J., Mostafa R., Banafsheh N., Masoud A. (2018). First principles study of a heavily nitrogen-doped (10, 0) carbon nanotube. *Physica E: Low-Dimensional Systems and Nanostructures*.

[10] Carroll D. L., Redlich P., Blase X. (1998). Effects of nanodomain formation on the electronic structure of doped carbon nanotubes. *Physical Review Letters*.

[11] Terrones M., Hsu W. K., Schilder A. (1998). Novel nanotubes and encapsulated nanowires. *Applied Physics A: Materials Science & Processing*.

[12] Kurt R., Karimi A. (2001). Influence of nitrogen on the growth mechanism of decorated C:N nanotubes. *ChemPhysChem*.

[13] Hsu W. K., Firth S., Redlich P. (2000). Boron-doping effects in carbon nanotubes. *Journal of Materials Chemistry*.

[14] Golberg D., Bando Y., Kurashima K., Sato T. (2001). Nanotubes of boron nitride filled with molybdenum clusters. *Journal of Nanoscience and Nanotechnology*.

[15] Redlich P., Loeffler J., Ajayan P. M., Bill J., Aldinger F., Rühle M. (1996). BCN nanotubes and boron doping of carbon nanotubes. *Chemical Physics Letters*.

[16] El-Sawy A. M., Mosa I. M., Su D. (2016). Controlling the active sites of sulfur-doped carbon nanotubegraphenenanolobes for highly efficient oxygen evolution and reduction catalysis. *Advanced Energy Materials*.

[17] Sharma P. P., Wu J., Yadav R. M. (2015). Nitrogen-doped carbon nanotube arrays for high-efficiency electrochemical reduction of CO_2_: on the understanding of defects, defect density, and selectivity. *Angewandte Chemie International Edition*.

[18] Tang J., Farmer D., Bangsaruntip S., Chiu K. C., Kumar B., Han S. J. Contact engineering and channel doping for robust carbon nanotube NFETs.

[19] Wang H., Maiyalagan T., Wang X. (2012). Review on recent progress in nitrogen-doped graphene: synthesis, characterization, and its potential applications. *ACS Catalysis*.

[20] He Y., Xu P., Zhang B. (2017). Ultrasmall MnO nanoparticles supported on nitrogen-doped carbon nanotubes as efficient anode materials for sodium ion batteries. *ACS Applied Materials & Interfaces*.

[21] He Y., Han X., Du Y. (2018). Conjugated polymer-mediated synthesis of sulfur- and nitrogen-doped carbon nanotubes as efficient anode materials for sodium ion batteries. *Nano Research*.

[22] Jang Y.-T., Ahn J.-H., Lee Y.-H., Ju B.-K. (2003). Effect of NH_3_ and thickness of catalyst on growth of carbon nanotubes using thermal chemical vapor deposition. *Chemical Physics Letters*.

[23] Lee Y. T., Kim N. S., Bae S. Y. (2003). Growth of vertically aligned nitrogen-doped carbon nanotubes: control of the nitrogen content over the temperature range 900–1100°C. *The Journal of Physical Chemistry B*.

[24] Moisala A., Nasibulin A. G., Brown D. P., Jiang H., Khriachtchev L., Kauppinen E. I. (2006). Single-walled carbon nanotube synthesis using ferrocene and iron pentacarbonyl in a laminar flow reactor. *Chemical Engineering Science*.

[25] Belz T., Baue A., Find J. (1998). Structural and chemical characterization of N-doped nanocarbons. *Carbon*.

[26] Esconjauregui S., Bhardwaj S., Yang J. (2014). Carbon nanotube growth on conductors: influence of the support structure and catalyst thickness. *Carbon*.

[27] Ionescu M. I., Zhang Y., Li R., Abou-Rachid H., Sun X. (2012). Nitrogen-doping effects on the growth, structure and electrical performance of carbon nanotubes obtained by spray pyrolysis method. *Applied Surface Science*.

[28] Sen R., Satishkumar B. C., Govindaraj A. (1998). B-C-N, C-N and B-N nanotubes produced by the pyrolysis of precursor molecules over Co catalysts. *Chemical Physics Letters*.

[29] He D., Jiang Y., Lv H., Pan M., Mu S. (2013). Nitrogen-doped reduced graphene oxide supports for noble metal catalysts with greatly enhanced activity and stability. *Applied Catalysis B: Environmental*.

[30] Matter P., Zhang L., Ozkan U. (2006). The role of nanostructure in nitrogen-containing carbon catalysts for the oxygen reduction reaction. *Journal of Catalysis*.

[31] Merzougui B., Hachimi A., Akinpelu A., Bukola S., Shao M. (2013). A Pt-free catalyst for oxygen reduction reaction based on Fe-N multiwalled carbon nanotube composites. *Electrochimica Acta*.

[32] Cao Y., Yu H., Tan J. (2013). Nitrogen-, phosphorous- and boron-doped carbon nanotubes as catalysts for the aerobic oxidation of cyclohexane. *Carbon*.

[33] Chen L., Xia K., Huang L., Li L., Pei L., Fei S. (2013). Facile synthesis and hydrogen storage application of nitrogen-doped carbon nanotubes with bamboo-like structure. *International Journal of Hydrogen Energy*.

[34] Vikkisk M., Kruusenberg I., Joost U., Shulga E., Tammeveski K. (2013). Electrocatalysis of oxygen reduction on nitrogen-containing multi-walled carbon nanotube modified glassy carbon electrodes. *Electrochimica Acta*.

[35] Yu C., Wang Y., Liu Y., Guo C., Hu Y. (2013). Facile growth of ZnO nanocrystals on nitrogen-doped carbon nanotubes for visible-light photodegradation of dyes. *Materials Letters*.

[36] Maldonado S., Morin S., Stevenson K. J. (2006). Structure, composition, and chemical reactivity of carbon nanotubes by selective nitrogen doping. *Carbon*.

[37] Fedi F., Domanov O., Ayala P., Pichler T. (2015). Raman and XPS analysis of pristine and annealed N-doped double-walled carbon nanotubes. *Physica Status Solidi*.

[38] Chen M., Zhao G., Shao L.-L. (2017). Controlled synthesis of nickel encapsulated into nitrogen-doped carbon nanotubes with covalent bonded interfaces: the structural and electronic modulation strategy for an efficient electrocatalyst in dye-sensitized solar cells. *Chemistry of Materials*.

[39] Rahmani A., Sauvajol J. L., Rols S., Benoit C. (2002). Nonresonant Raman spectrum in infinite and finite single-wall carbon nanotubes. *Physical Review B*.

[40] Inagaki M., Toyoda M., Soneda Y., Morishita T. (2018). Nitrogen-doped carbon materials. *Carbon*.

[41] Dresselhaus M. S., Eklund P. C. (2000). Phonons in carbon nanotubes. *Advances in Physics*.

[42] Saito R., Takeya T., Kimura T., Dresselhaus G., Dresselhaus M. S. (1998). Raman intensity of single-wall carbon nanotubes. *Physical Review B*.

[43] Alvarez L., Righi A., Guillard T. (2000). Resonant Raman study of the structure and electronic properties of single-wall carbon nanotubes. *Chemical Physics Letters*.

[44] Fuentes G. G., Borowiak-Palen E., Knupfer M. (2004). Formation and electronic properties of BC_3_ single-wall nanotubes upon boron substitution of carbon nanotubes. *Physical Review B*.

[45] Blase X., Benedict L. X., Shirley E. L., Louie S. G. (1994). Hybridization effects and metallicity in small radius carbon nanotubes. *Physical Review Letters*.

[46] Koretsune T., Saito S. (2008). Electronic structures and three-dimensional effects of boron-doped carbon nanotubes. *Science and Technology of Advanced Materials*.

[47] Kürti J., Zólyomi V., Kertesz M., Sun G., Baughman R. H., Kuzmany H. (2004). Individualities and average behavior in the physical properties of small diameter single-walled carbon nanotubes. *Carbon*.

[48] Miyamoto Y., Rubio A., Louie S. G., Cohen M. L. (1994). Electronic properties of tubule forms of hexagonal BC3. *Physical Review B*.

[49] Paraknowitsch J. P., Thomas A. (2013). Doping carbons beyond nitrogen: an overview of advanced heteroatom doped carbons with boron, sulphur and phosphorus for energy applications. *Energy & Environmental Science*.

[50] Yao Y., Zhang B., Shi J., Yang Q. (2015). Preparation of nitrogen-doped carbon nanotubes with different morphologies from melamine-formaldehyde resin. *ACS Applied Materials & Interfaces*.

[51] Kang J., Duan X., Wang C. (2018). Nitrogen-doped bamboo-like carbon nanotubes with Ni encapsulation for persulfate activation to remove emerging contaminants with excellent catalytic stability. *Chemical Engineering Journal*.

[52] Benoit C., Royer E., Poussigue G. (1992). The spectral moments method. *Journal of Physics: Condensed Matter*.

[53] Zimmermann J., Pavone P., Cuniberti G. (2008). Vibrational modes and low-temperature thermal properties of graphene and carbon nanotubes: minimal force-constant model. *Physical Review B*.

[54] Soler J. M., Artacho E., Gale J. D. (2002). The SIESTA method for *ab initio* order-*N* materials simulation. *Journal of Physics: Condensed Matter*.

[55] Perdew J. P., Burke K., Ernzerhof M. (1996). Generalized gradient approximation made simple. *Physical Review Letters*.

[56] Cardona M., Güntherodt G. (1982). *Light Scattering in Solids II*.

[57] Bell R. J. (1976). *Methods in Computational Physics*.

[58] Guha S., Menéndez J., Page J. B., Adams G. B. (1996). Empirical bond polarizability model for fullerenes. *Physical Review B*.

[59] Hermet P., Izard N., Rahmani A., Ghosez P. (2006). Raman scattering in crystalline oligothiophenes: a comparison between density functional theory and bond polarizability model. *The Journal of Physical Chemistry B*.

[60] Rahmani A., Jund P., Benoit C., Jullien R. (2001). Numerical study of the dynamic properties of silica aerogels. *Journal of Physics: Condensed Matter*.

[61] Ariharan A., Viswanathan B., Nandhakumar V. (2018). Nitrogen-incorporated carbon nanotube derived from polystyrene and polypyrrole as hydrogen storage material. *International Journal of Hydrogen Energy*.

[62] Jorio A., Saito R., Hafner J. H. (2001). Structural (*n*, *m*) determination of isolated single-wall carbon nanotubes by resonant Raman scattering. *Physical Review Letters*.

